# Function analysis of 5′-UTR of the cellulosomal *xyl*-*doc* cluster in *Clostridium papyrosolvens*

**DOI:** 10.1186/s13068-018-1040-0

**Published:** 2018-02-16

**Authors:** Xia Zou, Zhenxing Ren, Na Wang, Yin Cheng, Yuanyuan Jiang, Yan Wang, Chenggang Xu

**Affiliations:** 1 Research Center for Harmful Algae and Marine Biology, College of Life Science and Technology, Jinan University, Guangzhou, 510632 Guangdong Province China; 2 Institute of Applied Chemistry, Shanxi University, Taiyuan, 030006 Shanxi Province China; 3 Key Laboratory of Chemical Biology and Molecular Engineering of Ministry of Education, Institute of Biotechnology, Shanxi University, Taiyuan, 030006 Shanxi Province China; 4 Single-Cell Center, CAS Key Laboratory of Biofuels and Shandong Key Laboratory of Energy Genetics, Qingdao Institute of BioEnergy and BioProcess Technology, Chinese Academy of Sciences, Qingdao, 266101 Shandong Province China

**Keywords:** *Clostridium papyrosolvens*, Electrotransformation, *xyl*-*doc* gene cluster, Promoter, 5′ untranslated region (5′-UTR)

## Abstract

**Background:**

Anaerobic, mesophilic, and cellulolytic *Clostridium papyrosolvens* produces an efficient cellulolytic extracellular complex named cellulosome that hydrolyzes plant cell wall polysaccharides into simple sugars. Its genome harbors two long cellulosomal clusters: *cip*-*cel* operon encoding major cellulosome components (including scaffolding) and *xyl*-*doc* gene cluster encoding hemicellulases. Compared with works on *cip*-*cel* operon, there are much fewer studies on *xyl*-*doc* mainly due to its rare location in cellulolytic clostridia. Sequence analysis of *xyl*-*doc* revealed that it harbors a 5′ untranslated region (5′-UTR) which potentially plays a role in the regulation of downstream gene expression. Here, we analyzed the function of 5′-UTR of *xyl*-*doc* cluster in *C. papyrosolvens* in vivo via transformation technology developed in this study.

**Results:**

In this study, we firstly developed an electrotransformation method for *C. papyrosolvens* DSM 2782 before the analysis of 5′-UTR of *xyl*-*doc* cluster. In the optimized condition, a field with an intensity of 7.5–9.0 kV/cm was applied to a cuvette (0.2 cm gap) containing a mixture of plasmid and late cell suspended in exponential phase to form a 5 ms pulse in a sucrose-containing buffer. Afterwards, the putative promoter and the 5′-UTR of *xyl*-*doc* cluster were determined by sequence alignment. It is indicated that *xyl*-*doc* possesses a long conservative 5′-UTR with a complex secondary structure encompassing at least two perfect stem-loops which are potential candidates for controlling the transcriptional termination. In the last step, we employed an oxygen-independent flavin-based fluorescent protein (FbFP) as a quantitative reporter to analyze promoter activity and 5′-UTR function in vivo. It revealed that 5′-UTR significantly blocked transcription of downstream genes, but corn stover can relieve its suppression.

**Conclusions:**

In the present study, our results demonstrated that 5′-UTR of the cellulosomal *xyl*-*doc* cluster blocks the transcriptional activity of promoter. However, some substrates, such as corn stover, can relieve the effect of depression of 5′-UTR. Thus, it is speculated that 5′-UTR of *xyl*-*doc* was a putative riboswitch to regulate the expression of downstream cellulosomal genes, which is helpful to understand the complex regulation of cellulosome.

**Electronic supplementary material:**

The online version of this article (10.1186/s13068-018-1040-0) contains supplementary material, which is available to authorized users.

## Background

Cellulolytic clostridia are industrially significant microorganisms with a great capacity for producing renewable green chemicals from lignocellulosic biomass [1]. These anaerobes digest cellulose via extracellular enzymatic complex called cellulosome [2], which consist of a non-catalytic macromolecular scaffold and enzymes (including glycoside hydrolases, carbohydrate esterases, and polysaccharide lyases) [3]. The various enzymatic subunits are integrated by the scaffolding through cohesin–dockerin interaction in the complex [4]. These specific characteristics allow cellulosome to degrade cellulose substrate effectively.

**Fig. 1:**
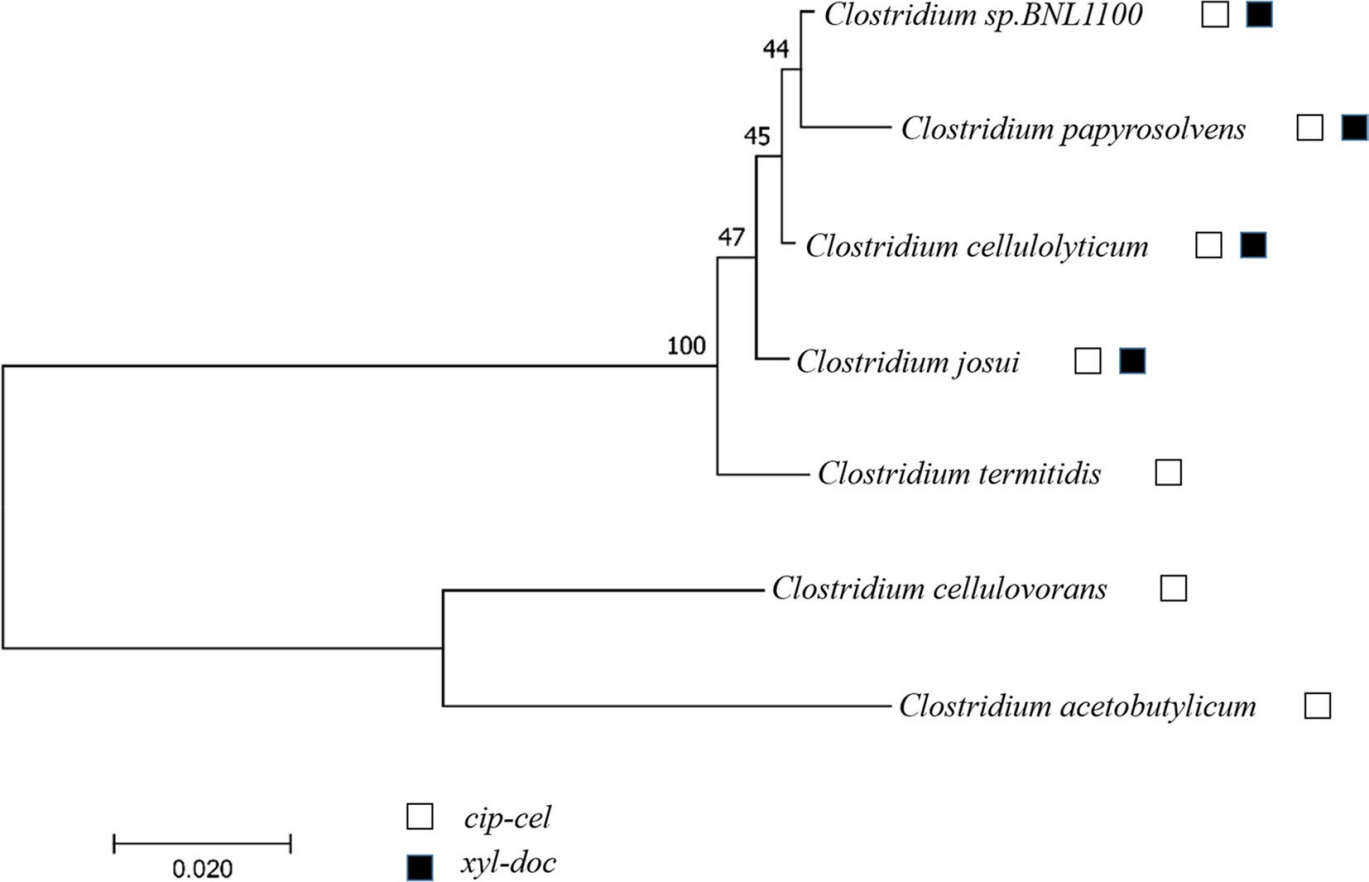
Phylogenetic analysis of mesophilic cellulolytic *Clostridium* spp. by Maximum Likelihood method. The percentage of trees in which the associated taxa clustered together is shown next to branches. Species harboring *cip*-*cel* cluster and *xyl*-*doc* clusters were labeled by open and filled box, respectively. Evolutionary analyses were conducted in MEGA7 [40]

Main components of cellulosome were encoded by two large gene clusters in *Clostridium cellulolyticum*, *cip*-*cel* [5, 6] and *xyl*-*do*c [7]. *cip*-*cel* operon encoding major cellulosomal components (including characterized cellulases and scaffolding protein) is essential for cellulose degradation. The *cip*-*cel* operon is regulated by carbon catabolite repression [8, 9], and the stoichiometry of its encoding cellulosomal components is controlled by the mechanism of selective RNA processing and stabilization [10]. In addition to *C. cellulolyticum*, orthologous *cip*-*cel* cluster was also found in many mesophilic *Clostridium* spp., such as *C. cellulovorans*, *C. acetobutylicum*, *C. termitidis*, *C. josui*, *C. sp* BNL1100, and *C. papyrosolvens* [10] (Fig. 1). Another large cluster, *xyl*-*doc,* encodes exclusively secreted dockerin-containing hemicellulase. Interestingly, enzymes encoded by *xyl*-*doc* are detected only in cellulosomes produced by cells grown on wheat straw-based medium [7, 11]. Hamza Celik et al. found out that the expression of *xyl*-*doc* in *C. cellulolyticum* was controlled by the upstream two-component system (TCS) XydS/R in response to straw [7]. However, only four clostridia (*C. cellulolyticum*, *C. josui*, *C. sp* BNL1100, and *C. papyrosolvens*) harbor *xyl*-*doc* cluster, which is less than the number of clostridia harboring *cip*-*cel* (Fig. 1). Meanwhile, original species of cellulolytic clostridia do not possess *xyl*-*doc*. Thus, *xyl*-*doc* evolves later than *cip*-*cel*.

Our previous transcriptomics study indicated that *xyl*-*doc* gene cluster harbors a 5′-untranslated region (5′-UTR) which might play a key role in regulation of downstream cellulosomal genes expression [9]. To investigate the function of 5′-UTR from *xyl*-*doc* cluster, *C. papyrosolvens* DSM 2782, essentially the most derived species among cellulolytic clostridia (Fig. 1), was employed in our study. For the first time, an electrotransformation method based upon optimized condition was developed in *C. papyrosolvens*. By means of this newly invented method, the putative promoter and 5′-UTR of *xyl*-*doc* cluster were characterized in vivo. The result indicated that 5′-UTR significantly blocked transcription activity of P*xyl*, whereas corn stover can slightly relieve its suppression. It is speculated that 5′-UTR of *xyl*-*doc* is a putative riboswitch to regulate the expression of downstream cellulosomal genes, which is helpful to understand the complex regulation of cellulosome.

## Results

### Restriction and modification systems in *C. papyrosolvens*

To develop a *C. papyrosolvens* transformation protocol, the restriction and modification (RM) systems and antibiotic sensitivity for *C. papyrosolvens* were investigated. Six putative RM operons were found by analyzing its genomic sequences (GenBank: ACXX00000000.2) in silico on REBASE (Restriction Enzyme Database) [12, 13], consisting of three putative methyltransferases (MT, type II) and 3 putative restriction endonuclease (RE, type IV). Through protein BLAST in PubMed (http://blast.ncbi.nlm.nih.gov/), enzymes in *C. papyrosolvens* RM systems with their hypothetical specificities or functions were predicted (Additional file 1: Table S1). Two methyltransferases, M.Cpa2782ORF3030P and M.Cpa2782ORF3889P, have homologues in *C. cellulolyticum*, but no genes are homologous to *Msp*I and M.*Msp*I (the main RM system in *C. cellulolyticum* H10).

**Fig. 2:**
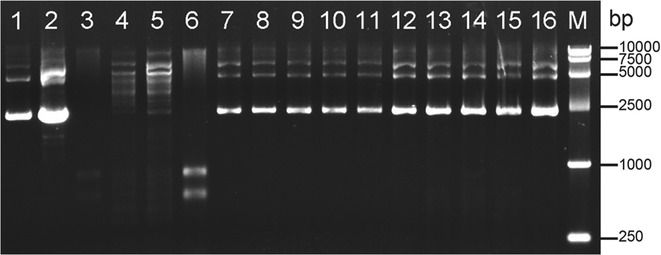
Characterization of the restriction profile by restriction assay. The plasmids (pMTC6) extracted from JM110 (Dam −, Dcm −) and Trans 5α (Dam +, Dcm +) were respectively incubated with *C. papyrosolvens* cell lysate in various NEB buffers for 20 h at 37 °C, using *C. cellulolyticum* cell lysate as a control. Lane 1 and 2: pMTC6 extracted from JM110 and DH5α; lane 3: *C. cellulolyticum* cell lysate; Lane 4 and 5: pMTC6 (Dam −, Dcm −) and pMTC6 (Dam +, Dcm +) incubated with *C. cellulolyticum* cell lysate; lane 6: *C. papyrosolvens* cell lysate; lane 7–11: pMTC6 (Dam −, Dcm −) incubated with *C. papyrosolvens* cell lysate in NEB buffer 1, 2, 3, 4, and Cutsmart buffer; lane 12–16: pMTC6 (Dam +, Dcm +) incubated with *C. papyrosolvens* cell lysate in various NEB buffers

Furthermore, the restriction profile of *C. papyrosolvens* DSM 2782 was analyzed by incubating its cell lysate with *E. coli*-*Clostridium* shuttle plasmid pMTC6 [10]. The plasmid DNA showed no change after incubating with the whole cell extract of *C. papyrosol*vens DSM 2782 in various NEB buffers, but had been digested by that of *C. cellulolyticum* H10 because of its endogenous *Msp*I (Fig. 2). The same results were obtained for pMTC6 isolated from *E. coli* Trans 5α (dam + , dcm +) and JM110 (dam −, dcm −) (Fig. 2). We therefore speculated that *C. papyrosolvens* DSM 2782 does not harbor any restriction endonucleases to digest exogenous DNA.

### Development of electrotransformation method for *C. papyrosolvens*

It is necessary to carry out antibiotic sensitivity test to find an appropriate selection marker for screening *C. papyrosolvens* transformants. The antibiotic sensitivity of *C. papyrosolvens* was tested with nine antibiotics at different dilutions (Additional file 2: Figure S1a). The 90% inhibitory concentration (IC90) was used to reflect the antibiotic sensitivities. *C. papyrosolvens* is most sensitive to erythromycin, lincomycin, and tetracycline with the lowest IC90 (0.06, 0.06, and 0.15 µg/ml, respectively) among all nine antibiotics (Additional file 2: Figure S1b). Therefore, resistance genes for erythromycin, lincomycin, and tetracycline are appropriate as selected markers to screen the transformants of *C. papyrosolvens*.

*Escherichia coli*-*Clostridium* shuttle plasmid pMTC6 harbors erythromycin resistance gene MLS and pIM13 replicon from *Bacillus subtilis*, derivatives of which have been transformed into many *Clostridium*, such as *C. cellulolyticum* [14] and *C. acetobutylicum* [15]. Thus, it has been used to transform *C. papyrosolvens* in this research. We conducted a series of pilot experiments based on the previous description of the transfer of foreign DNA to other clostridial species (*C. cellulolyticum* [16], *C. acetobutylicum* [17], *C. saccharoperbutylacetonicum* [18], and *C. thermocellum* [19]). Electrotransformation conditions were further investigated and optimized in terms of growth states of cells (OD600 = 0.4–1.6), electroporation buffers (SMP, HSM and SMG), cuvettes (0.2 and 0.4 cm gap), and electrical parameters (field strength 6.0–10 kV/cm, 200/400 Ω, 25 μF). Transformants were successfully obtained under certain conditions (Exponential late cells were resuspended in a sucrose-containing buffer, transferred in 0.2 cm cuvettes, a field strength of 7.5–9.0 kV/cm was applied to get a 5 ms pulse), but with a very low transformation efficiency of 1–5 CFU/μg DNA. The transformation efficiency increased to about 20 CFU/μg DNA when 2.5 mg/ml glycine was added.

**Fig. 3:**
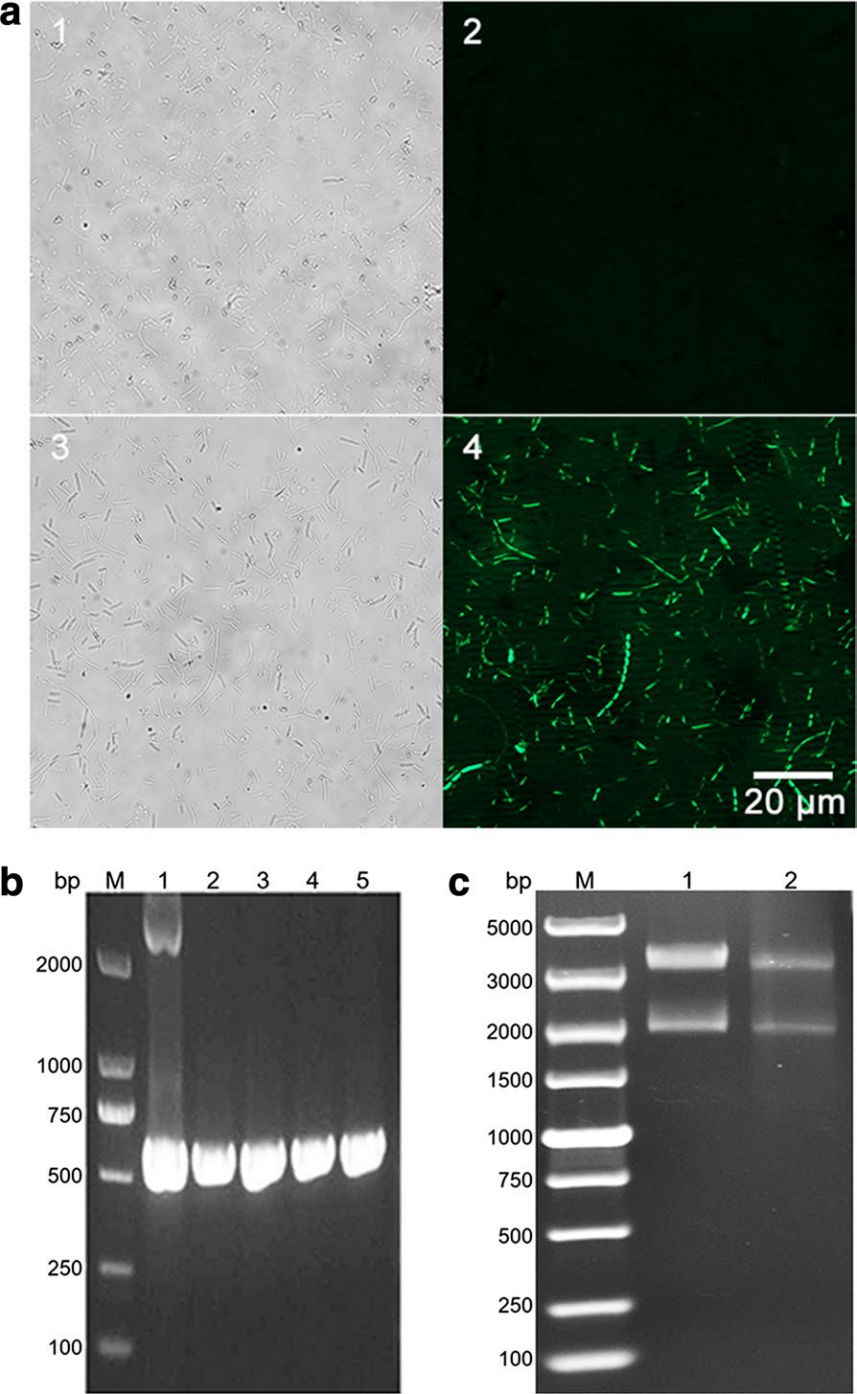
Identification of *C. papyrosolvens* transformants. **a** The phase contrast pictures of the wild-type *C. papyrosolvens* (**1**, **2**) and the transformants pMTC6 (**3**, **4**) observed by fluorescence microscope at ×400 magnification. **b** *fbfp* gene was amplified by PCR using transformant colonies as templates (lanes 2, 3, 4, and 5) or pMTC6 as a control (lane 1). **c** Gel electrophoresis showing *Hin*dIII digestion profile of the pMTC6 plasmid extracted from *E. coli* (control; lane 1) and the pMTC6 plasmid extracted from *C. papyrosolvens* transformants (lane2)

pMTC6 harbors a fluorescence protein gene (*fbfp*) from *Pseudomonas putida* encoding anaerobic fluorescent protein driven by the thiolase gene promoter (P*thl*, a promoter from *C. acetobutylicum*) (Ex 452 nm; Em 495 nm) [20, 21]. To confirm successful transfer of the plasmid DNA into cells, we observed cells fluorescence via a fluorescence microscope (BX51, Olympus, Japan) to obtain phase contrast pictures (Fig. 3a). Wild-type *C. papyrosolvens* did not show any fluorescence, but the transformants of pMTC6 could emit green fluorescence. Moreover, transformants were examined by detecting the *fbfp* gene using PCR analysis. As shown in Fig. 3b, fragments with the expected size of about 500 bp were obtained from presumptive transformants, as well as from a positive control. Furthermore, the presence of pMTC6 in erythromycin-resistant colonies was verified by its isolation and restriction digestion by *Hin*dIII (Fig. 3c). Bands of the digested DNA were compared to bands of pMTC6 isolated from *E. coli* in the same way, indicating that they have a similar digestion pattern (Fig. 3c). All the above approaches demonstrated that pMTC6 had been successfully transformed into *C. papyrosolvens* DSM 2782.

### Sequence analysis of upstream of the *xyl*-*doc* gene cluster

**Fig. 4:**
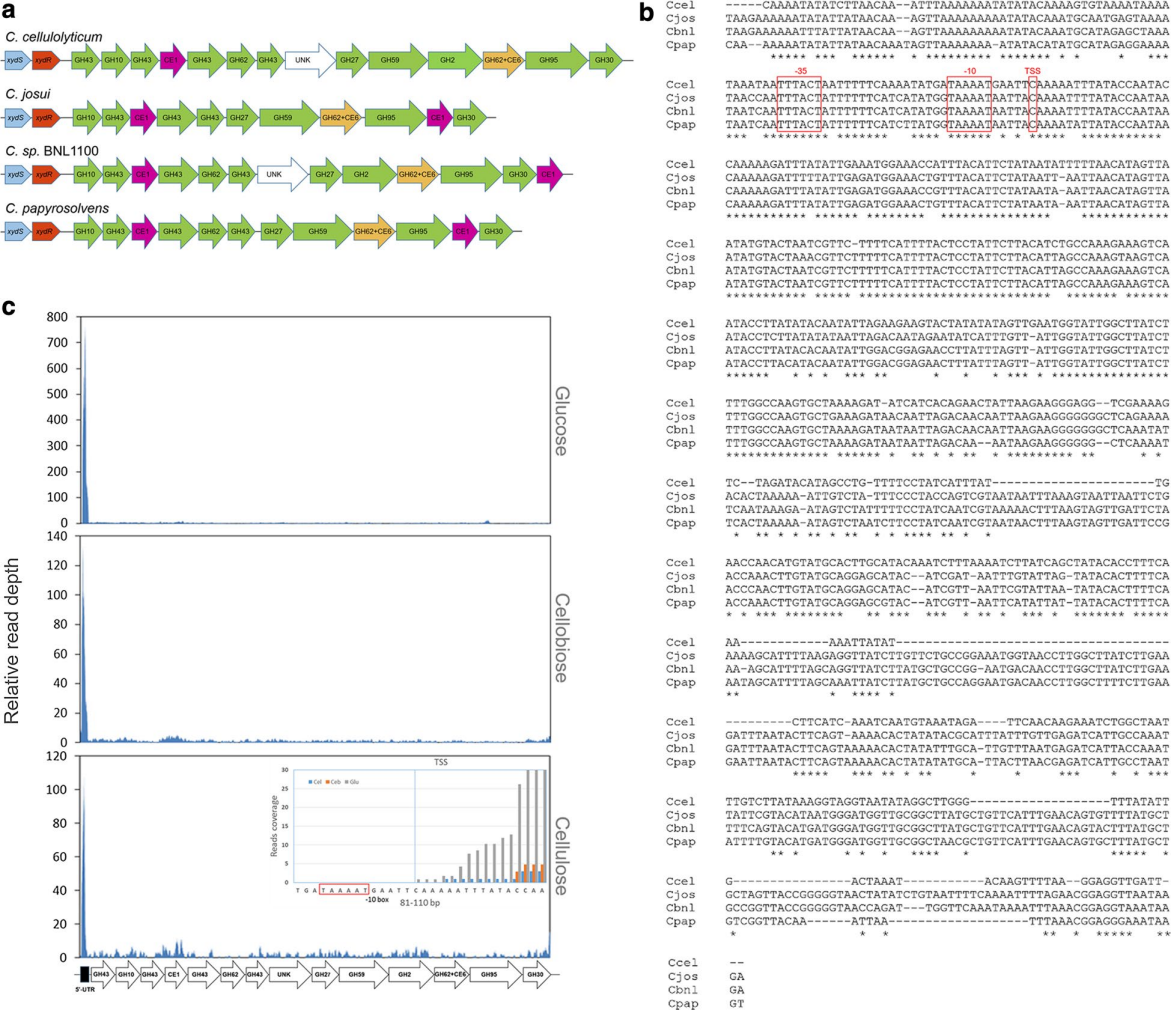
Sequence analysis of *xyl*-*doc* gene clusters from *C. cellulolyticum*, *C. josui*, *C. sp* BNL1100, and *C. papyrosolvens*. **a** Genetic organization of TCS-encoding *xyd*S/R and *xyl*-*doc* genes. The catalytic domains predicted glycoside-hydrolase (GHx) or carbohydrate esterase (CEx) family when known or UNK for a domain of unknown function are given. **b** Sequences alignment of intergenic region between TCS and *xyl*-*doc* from four *Clostridium* spp. Predicted − 35 and − 10 region and transcription start site (TSS) of promoters are labeled. **c** Transcriptional profile of the *xyl*-*doc* gene cluster upstream region of *C. cellulolyticum* (Data from our previous papers. Accession number in GEO of NCBI: GSE57652)

The *xyl*-*doc* gene clusters have been found in *C. cellulolyticum*, *C. josui*, *C. sp* BNL1100, and *C. papyrosolvens* (Fig. 4a). It has been reported that the *xyl*-*doc* cluster in *C. cellulolyticum* is controlled by its upstream TCS [7, 9]. Although the gene numbers of *xyl*-*doc* clusters in these four species vary from 11 to 14, nine orthologous genes are present in all *xyl*-*doc* clusters. Interestingly, at the beginning of *xyl*-*doc* in *C. cellulolyticum,* an additional gene encoding GH43 family enzyme is located in front of GH10-encoding gene which is the first gene of *xyl*-*doc* clusters in the three other species.

Sequences of the intergenic region between TCS and *xyl*-*doc* from the four *Clostridium* spp. were firstly aligned by ClustalW (Fig. 4b). It is showed that these four sequences are homologous between each other with above 85% identity. However, downstream 190 bp from *C. cellulolyticum* (*i.e*., upstream the first gene encoding GH43) differs from other species sequences suggesting that this particular 190 bp sequence may have been obtained through horizontal transfer together with its following GH43-encoding gene in *C. cellulolyticum*. Furthermore, a consensus promoter (named P*xyl*) for *xyl*-*doc* was predicted to be located at 65-bp upstream in the intergenic region by BPROM (http://www.softberry.com/), suggesting that P*xyl* will transcribe a long 5′-UTR (more than 400 bp) (Fig. 4b). The predicted results are consistent with our previous transcriptomic data in *C. cellulolyticum* (Fig. 4c). Transcription profiles of *xyl*-*doc* cluster indicated that transcriptional initiation of *xyl*-*doc* cluster occurred at predicted transcription start site (TSS). These results about *xyl*-*doc* promoter were also completely consistent with the previous results of electrophoretic mobility shift assay (EMSA) [7]. Furthermore, a free 5′-UTR with much higher transcriptional level than its downstream genes was transcribed under all three carbon sources (glucose, cellobiose, and cellulose) (Fig. 4c). However, 5′-UTR transcriptional level on cellulose was the lowest among all carbon sources, which is as few as 10–15% of that on glucose. On the other hand, the downstream genes of 5′-UTR were transcribed in extremely low levels especially on cellulose, which was also observed in translation level in previous study [7]. Unlike the previous explanation, we think, in addition to low activation state of TCS (XydS/R), transcription of *xyl*-*doc* especially on cellulose is repressed by 5′-UTR, in which there might be some transcription termination signals or cis-acting elements of a specific repressor.

**Fig. 5:**
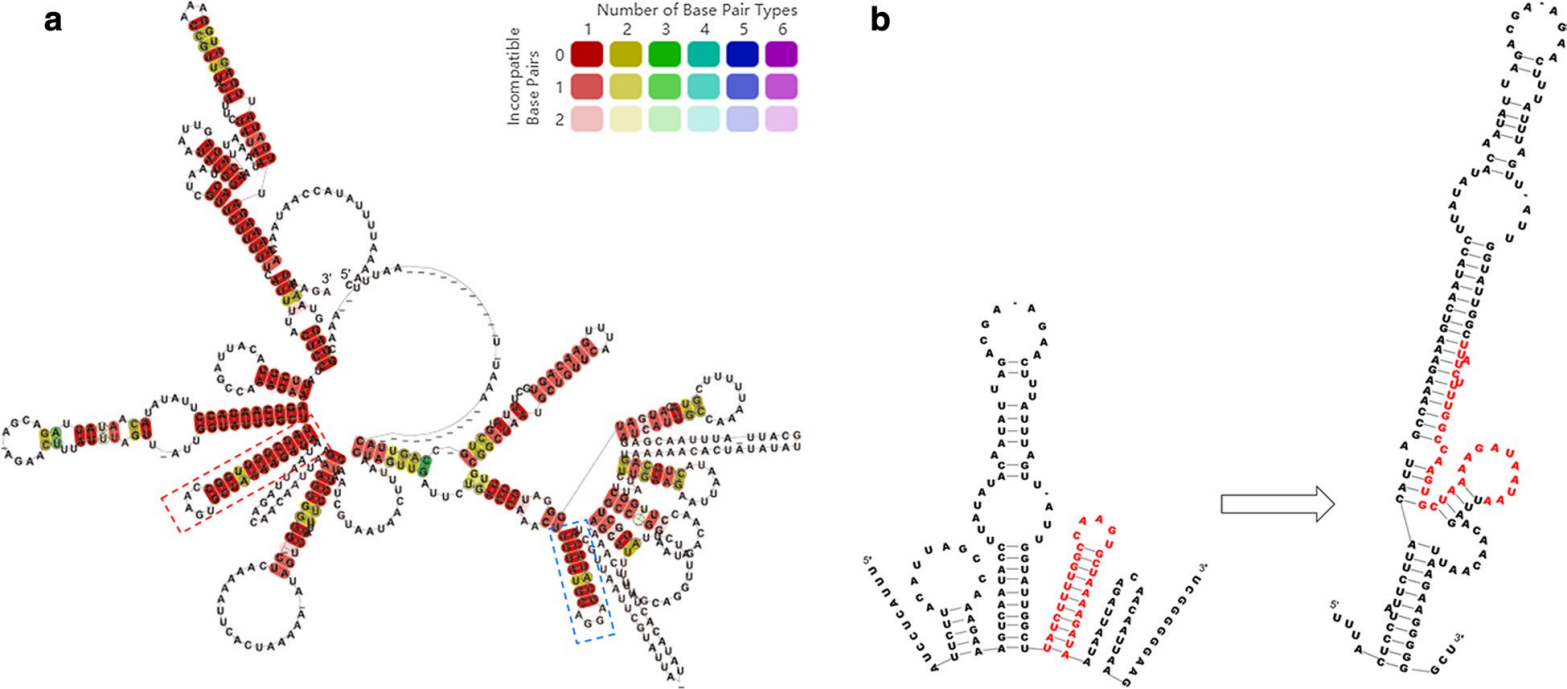
Secondary structure prediction of 5′-UTR. **a** Conservative secondary structure of 5′-UTR was predicted by the RNAalifold [41] option of the ViennaRNA package [42], in which there are two perfect stem-loop structures (dashed box) in the conservative region. **b** Conformation shift of the first stem-loop

To clarify the putative function of 5′-UTR to control transcription of its downstream genes, 5′-UTR consensus secondary structures were predicted by RNAalifold (http://rna.tbi.univie.ac.at/cgi-bin/RNAWebSuite/RNAalifold.cgi) (Fig. 5). It showed that 5′-UTR conservative sequences were folded into a complex structure, in which there were two perfect stem-loop structures (dashed box) that might be potential elements for premature termination (Fig. 5a). However, this structure of 5′-UTR can shift to another stable structure, in which the first stem-loop (red dashed box in Fig. 5a) would be opened and integrated into a large stem-loop structure (Fig. 5b). Thus, it is suggested that 5′-UTR of *xyl*-*doc* harbors a convertible structure of stem-loops, which can potentially control on and off of transcription of its downstream genes as expression platform of riboswitch.

### Function analysis of 5′-UTR of the *xyl*-*doc* gene cluster in vivo

**Fig. 6:**
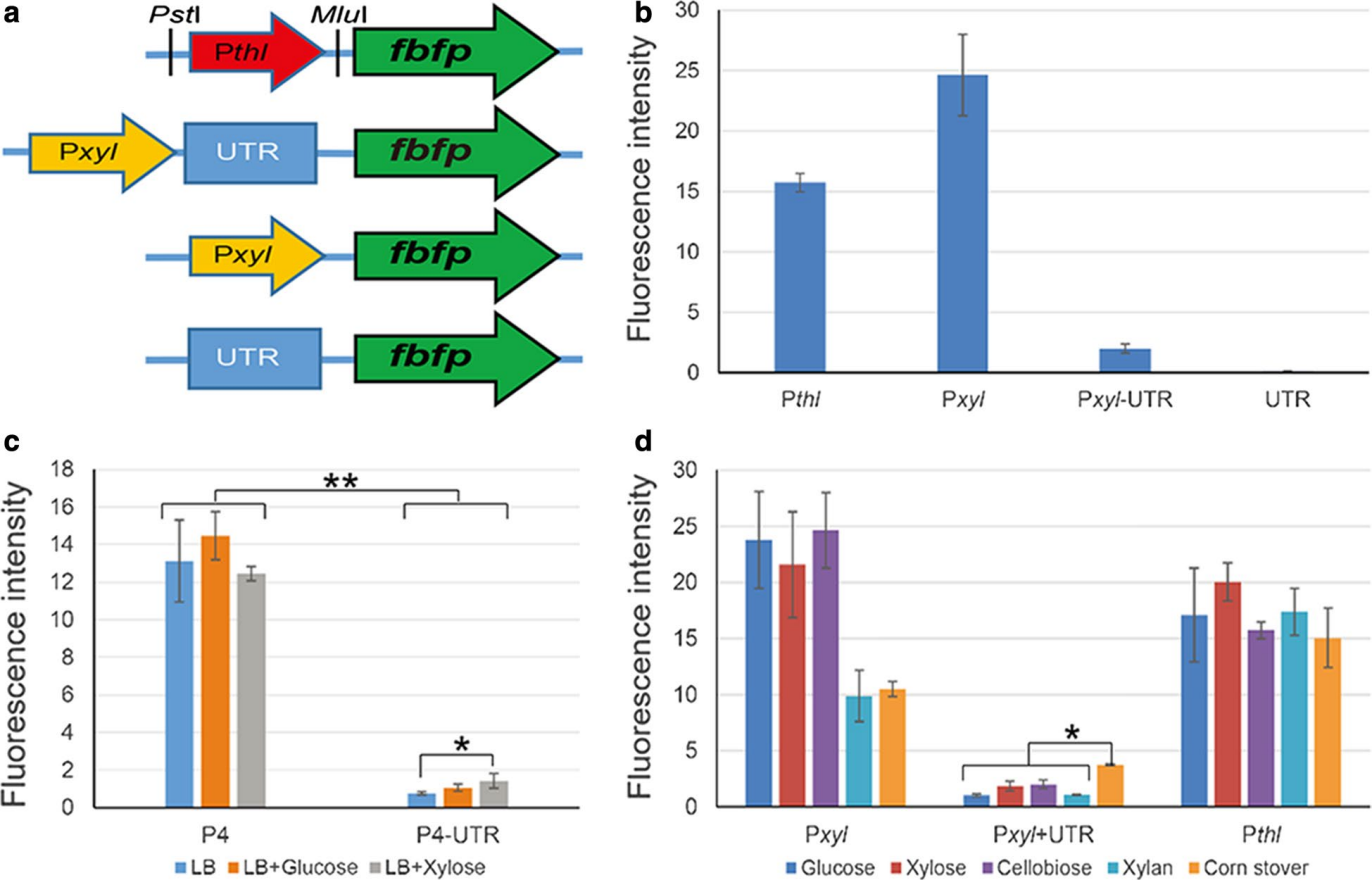
Function analysis of promoters and 5′-UTR of *xyl*-*doc*. **a** Various transcriptional fusions corresponding to different *xyl*-*doc* cluster upstream subregions were constructed by fusing promoter fragments to upstream of *fbfp* gene, and P*thl* promoter is as the control. **b** Activities of various fragments promoting expression of FbFP were measured fluorometrically in *C. papyrosolvens* grown on cellobiose. **c** Promoter activity of P4 and P4-UTR were compared in *E. coli* grown on LB medium supplemented with glucose or xylose. **d** Comparison of activities of P*xyl*, P*xyl*-UTR, and P*thl* in *C. papyrosolvens* grown on different carbon sources. Error bars indicate s.d. of mean of experiments in triplicate (*P < 0.05, **P < 0.01; Student’s *t* test)

To verify the function of 5′-UTR, the promoter activity of the *xyl*-*doc* cluster upstream sequence from *C. papyrosolvens* DSM 2782 was analyzed by employing the FbFP reporter system [21]. Firstly, P*thl* promoter of *fbfp* in pMTC6 was replaced by P*xyl*-UTR, P*xyl* promoter, or UTR sequences from the whole upstream sequence of *xyl*-*doc*, respectively, all of which harboring the same ribosome-binding sequence (Fig. 6a). These recombinant plasmids were transformed into *C. papyrosolvens* DSM 2782 using the above-developed transformation method. Their promoter activities were subsequently analyzed by comparing fluorescence intensity of FbFP reporter from transformants grown on cellobiose (Fig. 6b). The results indicated that P*xyl* had a promoter activity equivalent to 157% of P*thl* activity, whereas UTR had no promoter activity as expected. However, P*xyl*-UTR had hardly any promoter activity, suggesting that there is a premature transcription termination signal or repressor-binding site in UTR to prevent transcription activity of P*xyl* (Fig. 6b). This result of P*xyl*-UTR in *C. papyrosolvens* is consistent with the transcriptional profile of *xyl*-*doc* in *C. cellulolyticum*, in which transcription of the beginning of the first gene of *xyl*-*doc* was suddenly abolished after transcription occurrence of 5′-UTR (Fig. 4c).

Furthermore, in order to exclude the possibility of endogenous repressor effect to UTR transcription, UTR was promoted by a synthetic mini σ^A^ promoter P4 [22] and transcribed in the distant relative *E. coli* (Fig. 6c). The results indicated that UTR also significantly decreases the activity of P4 in *E. coli* (*P* value < 0.001; Student’s *t* test), in which P4-UTR activity was only 5.8% of P4 activity when *E. coli* cells grew on LB medium. Furthermore, when cells were grown on xylose-supplemented LB medium, the ratio of P4-UTR activity to P4 activity was significantly increased to 11.5% (*P* value < 0.05; Student’s *t* test) (Fig. 6c). Thus, it is revealed that the premature transcription termination of *xyl*-*doc* is caused by intrinsic sequence of UTR, but not trans-acting factor of *C. papyrosolvens*.

In order to further analyze the function of P*xyl* and UTR, the promoter activities of P*xyl* and P*xyl*-UTR were measured under various carbon sources (glucose, xylose, cellobiose, xylan, and corn stover). The result indicated that the promoter activities of P*xyl* were different for various carbon sources. The activities of P*xyl* on monosaccharide and disaccharide (glucose, xylose and cellobiose) were more than twice activities on polysaccharide (xylan and corn stover) (*P* value < 0.01; Student’s *t* test), while activities of P*thl* among all carbon sources did not change significantly (Fig. 6d). It is suggested that P*xyl* controlled by TCS XydS/R seems not being strictly induced by a certain type of substrates, such as hexose or pentose, monosaccharide or oligosaccharide, but induced by broad-spectrum carbon sources depending on concentration of their soluble sugars. On the other hand, P*xyl*-UTR has much less (< 10%) promoter activity than P*thl* on all carbon sources except corn stover, which is consistent with the previous report found in *C. cellulolyticum* [7]. The promoter activity of P*xyl*-UTR on corn stover was 25% of P*thl* activity (Fig. 6d). Thus, it is indicated that the first transcribed 5′-UTR by P*xyl* under control of XydR greatly reduced the promoter activity of P*xyl*, but corn stover could partly relieve suppression of UTR to P*xyl*, suggesting that degradation products derived from corn stover can potentially interact with UTR to shift its structure of premature transcription termination.

## Discussion

In nature, a lot of clostridia can degrade lignocellulose and ferment the degraded substance to produce hydrogen, ethanol, or butanol, which provides one more means to produce cellulosic biofuel via metabolic engineering. Currently, genetic transformation systems have been successfully established for many types of *Clostridium*, for example, *C. cellulolyticum* [23], *C. acetobutylicum* [24, 25], and *C. thermocellum* [26, 27]. In this work, we described the development of an electrotransformation protocol for genetic manipulation of *C. papyrosolvens* DSM 2782. Compared to other bacteria, transformation of *C. papyrosolvens* is relatively simple with no need of methylation, but the transformation efficiency is generally quite low. Cell wall-weakening agents, such as glycine and isoniazid, were able to improve transformation efficiency. When treated with 10 mg/ml of glycine, the transformation efficiency of *C. cellulolyticum* was improved for about 24-fold [28]. In this study, we demonstrated that adding 2.5 mg/ml glycine had slight positive effect on improvement of the electrotransformation efficiency of *C. papyrosolvens*.

The hard transformation of *C. papyrosolvens* may be caused by its special cell envelope. Compared to *C. cellulolyticum*, *C. papyrosolvens* cells were lacking small cellulosomal protuberances and were covered by a layer of viscous materials, which may be the extracellular polymeric substance (EPS) illustrated by the electron microscopy results (Additional file 3: Figure S2). We speculate that the discharged EPS on cell surface may be the main obstacle for foreign DNA transfer by electroporation [29]. It is noteworthy that published article by Ferdinand et al. shows that there is no protuberance on the surface of *C. cellulolyticum* [30], which is contrary to what we have observed. This difference might be caused by the difference in the composition of the culture media. Complete medium was used for strain growth in our study, whereas minimal medium was applied in the previous publication. On the other hand, cells with genetic competence can internalize exogenous DNA [31]. It has been reported that EPS can improve competent cells’ transformation efficiency by facilitating the binding plasmid DNA for cellular uptake [32, 33]. This competence has been developed for genetic transformation of many strains in laboratory cultures by coordinately regulated expression of gene sets encoding effectors of DNA transport and recombination, such as *Streptococcus sanguinis* [34] and *Streptococcus mutans* [35]. Thus, development of competence for genetic transformation of *C. papyrosolvens* is a complementary alternative.

In cellulolytic clostridia, cells sense extracellular sugars using TCS and control expression of relative cellulase genes [9]. We found that recognizing of polysaccharides, such as cellulose [36] and xylan [14], needs an additional sugar-binding protein associated with the TCS, thus forming the three-component system. We speculate that sensor histidine kinase of TCS can not directly recognize macromolecular oligosaccharides. However, expression of *xyl*-*doc* genes encoding hemicellulases is also controlled by the TCS XydS/R which does not contain the additional sugar-binding protein in response to hemicellulose-including straw [7]. Why do not cells employ the three-component system to recognize hemicelluloses? One possible cause is the composition difference of polysaccharides. It is known that cellulose and xylan are homogeneous polymers that respectively consist of glucose and xylose units, so cells can easily evolve the third specific protein of three-component system to recognize them. However, hemicelluloses belong to a highly heterogeneous group of noncellulosic polysaccharides, which may contain pentoses, hexoses, and/or uronic acids, resulting in the absence of a specific protein to directly recognize them. This hypothesis is perfectly compatible with the promoter activity of P*xyl* controlled by XydS/R (Fig. 6d), which can not specifically respond to hemicelluloses such as corn stover, though can be induced by those broad-spectrum carbon sources composed of high diversity of degradation products of hemicellulose.

In order to accurately recognize hemicelluloses, cells have to take alternatives, in which a 5′-UTR is transcribed in *xyl*-*doc* upstream region under P*xyl* controlled by XydS/R. 5′-UTR results in premature transcription termination of *xyl*-*doc* genes unless cells are grown on hemicelluloses, suggesting that 5′-UTR may be a riboswitch sensing intracellular oligosaccharides derived from hemicelluloses to regulate expression of the *xyl*-*doc* cluster. Thus, transcription of *xyl*-*doc* is the combined effect of both XydS/R and UTR regulation, but they are different in recognized signal sources. XydS/R mainly senses the extracellular molecular signal by extracytoplasmic sensing domains (most common domain is the PAS-lake domain) of the sensor histidine kinases [37], while UTR directly senses intracellular metabolites via its aptamer domains [38]. The dual control mode of TCS and riboswitch also found in the regulation of Mg^2+^ transporter MgtA from *Salmonella enteric* [39]. The TCS PhoP/Q responds to periplasmic Mg^2+^, governs *mgtA* transcription initiation at all investigated Mg^2+^ concentrations, and 5′-UTR of the *mgtA* gene controls transcription elongation into the *mgtA* coding region when cells are grown in media with < 50 mM Mg^2+^.

**Fig. 7:**
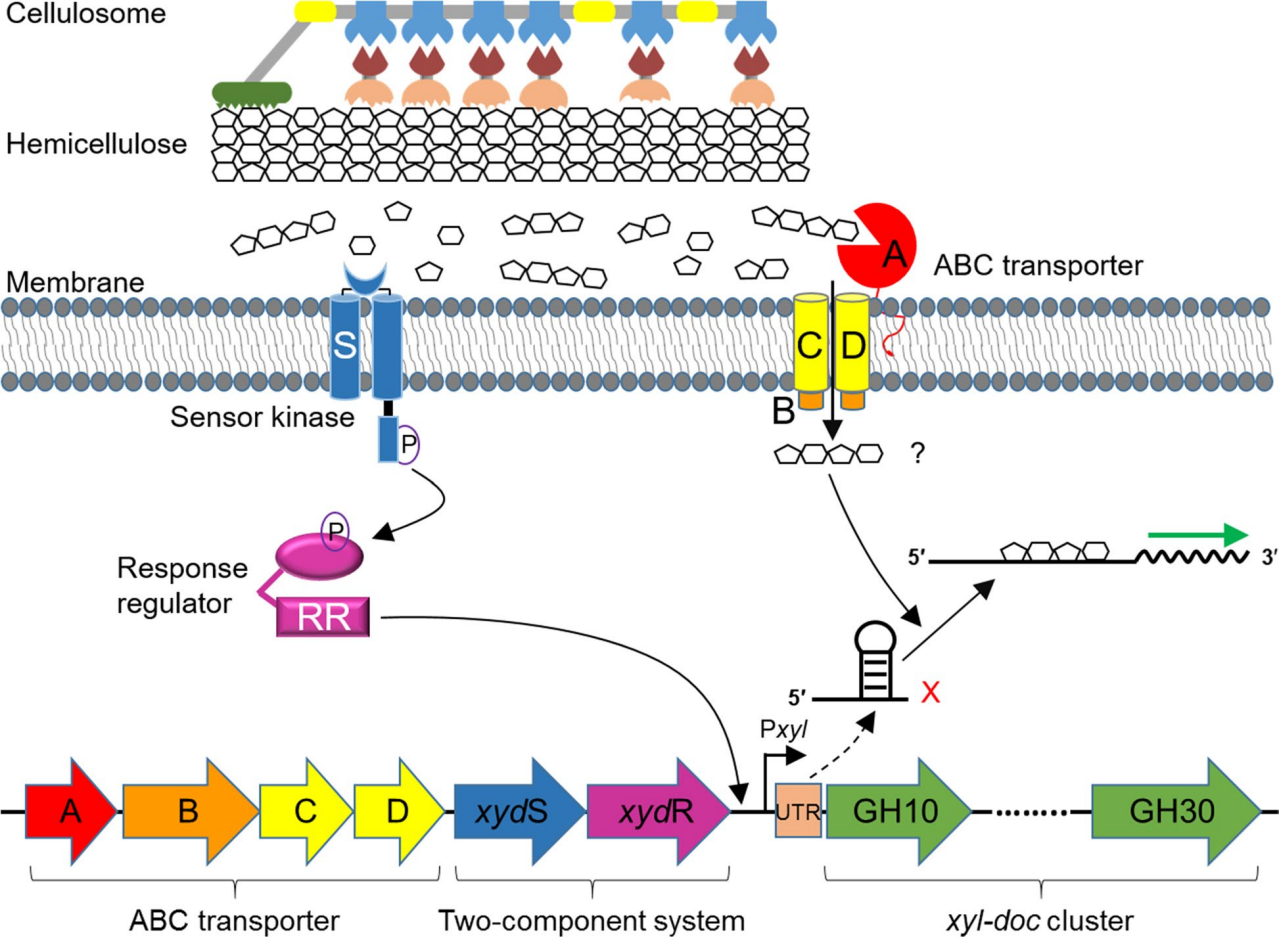
Model for expression and regulation of *xyl*-*doc* cluster in *C. papyrosolvens*. The cell employs TCS XydS/R to sense extracellular monosaccharides and controls 5′-UTR and ABC transporter. The ABC transporter captures extracellular oligosaccharides from the environment and transports them into cells. The transcribed 5′-UTR as riboswitch specifically recognizes the intracellular oligosaccharide signal resulted from hemicellulose hydrolysis and allows the transcription to proceed through the entire *xyl*-*doc* cluster, which would otherwise prematurely terminate the transcription

These findings allow us to propose a model for transcription control of *xyl*-*doc* cluster (Fig. 7). Firstly, the TCS XydS/R responds to availability of extracellular soluble saccharides resulted from lignocellulose hydrolysis, especially monosaccharides derived from hemicellulose, which promotes the transcription initiation of 5′-UTR and ABC transporter. ABC transporters then transport extracellular soluble sugars into cells. Finally, the riboswitches located in transcribed 5′-UTR can specifically recognize the intracellular soluble oligosaccharide signal resulted from hemicellulose hydrolysis and allow the transcription to proceed through the entire *xyl*-*doc* cluster, which would otherwise prematurely terminate the transcription. This work has contributed to understanding the complex regulation of *xyl*-*doc* cluster. The future work will be identification of the signal molecules involved and study of the mechanism of premature termination. Exploring riboswitch regulatory mechanism of the *xyl*-*doc* cluster is helpful to understand the expression of cellulosome. Such knowledge will facilitate robust and green conversion of lignocellulose to valuable products.

## Conclusions

In this study, a unique electrotransformation method for *C. papyrosolvens* DSM 2782 was developed for the first time, followed by the analysis of antibiotic sensitivity and the restriction modification (RM) systems, The method was further employed to obtain several *C. papyrosolvens* transformants for the analysis of promoter activity of *xyl*-*doc* cluster and the function of 5′-UTR. Our results demonstrated that 5′-UTR of the cellulosomal *xyl*-*doc* cluster blocks the transcriptional activity of promoter. However, some substrates, such as corn stover, can relieve the depression of 5′-UTR. Thus, it is speculated that 5′-UTR of *xyl*-*doc* is a putative riboswitch to regulate the expression of downstream cellulosomal genes. These features represent a distinct environment-sensing strategy for hemicellulase expression, which can be exploited for processing and genetic engineering of microbial cellulolysis.

## Methods

### Strains and growth conditions

*Escherichia coli* was used as the host strain for routine cloning and incubated at 37 °C in Luria–Bertani (LB) medium. *C. papyrosolvens* and its derivatives were anaerobically cultured at 35 °C in GS-2 medium (K_2_HPO_4_ 2.9 g/L, KH_2_PO_4_ 1.5 g/L, Urea 2.1 g/L, resazurin 1.0 mg/L, yeast extract 6.0 g/L, Cysteine-HCl 0.5 g/L, MOPS 10.0 g/L, Trisodium citrate 3.0 g/L, pH 7.4) supplemented with 3.0 g/L of cellobiose as the sole carbon source (default carbon source unless otherwise stated) [28]. In promoter activity assay, glucose, xylose, xylan, or corn stover (3.0 g/L) was used instead of cellobiose as carbon sources, respectively. The medium was deoxygenized in the anaerobic chamber using resazurin (0.0005%) as the indicator and sterilized at 121 °C for 20 min. A shuttle vector, pMTC6, which contains *fbfp* gene encoding FbFp, was used for detection and quantification of promoter activity [28]. When required, the media for *E. coli* and *C. papyrosolvens* were supplemented with 100 μg/ml ampicillin or 20 μg/ml erythromycin.

### Antibiotics screening

Absorbance at 600 nm (A600) of *C. papyrosolvens* culture was measured using spectrometer when cultured for 24 h under nine types of antibiotics (ampicillin, apramycin, chloromycetin, erythromycin, hygromycin, kanamycin, lincomycin, spectinomycin, and tetracycline) with different concentrations. All experiments were performed in triplicates. The relationships between the inhibitory rates and antibiotic concentrations were fitted by the exponential rise equation $\left( {f = a*\left( {1 - b^{\wedge}{\text{x}}} \right)} \right)$. The 90% inhibitory concentration (IC90) was obtained by fitting curve to represent the antibiotic sensitivity.

### Fluorescent microscopy

Five milliliters of *C. papyrosolvens* or its transformants were harvested by centrifugation after culturing for about 20 h, washed twice, and resuspended in 200 μl of distilled water. Resuspended cells (2 μl) were detected with a BX51 fluorescent microscope (Olympus, Japan).

### DNA manipulation

Isolation and manipulation of recombinant DNA were performed using standard techniques. DNA was amplified by PCR using synthetic oligonucleotide primers and Pfu DNA polymerase (TransGen Biotech, China) (the PCR primers are listed in Additional file 4: Table S2). PCR products of putative promoter (named P*xyl*), 5′-UTR and the full upstream region (named P*xyl*-UTR) of *xyl*-*doc* cluster were purified and digested with restriction enzymes *Pst*I and *Mlu*I. Then the digested fragments were respectively ligated to the shuttle vector pMTC6 which was digested with the same enzymes, resulting in the replacement of the original P*thl* promoter (promoter for *fbfp* gene in pMTC6).

### Characterization of the restriction system

Crude extract of *C. papyrosolvens* was prepared from 5 ml late-exponential-phase culture (OD600 = 0.8–1.0) using the high-throughput tissue grinder (QIAGEN, Germany) with 0.1–0.2-mm glass beads. For restriction assays, 500 ng DNA substrates were mixed with 10 μg crude extract and incubated at 37 °C for 20 h in various NEB buffers. The products were analyzed by electrophoresis through agarose.

### Transformation procedures

For all electroporation experiments, an electroporator (ECM630, BTX, USA) was used. Electroporation was performed and optimized based on the previously described method [16]. *C. papyrosolvens* was grown for 17–24 h in 50 ml cultures in GS medium to late exponential phase (OD600 = 0.8–1.0). The cell wall-weakening agents glycine (0–40 mg/ml), threonine (0–40 mg/ml), isoniacin (0–40 μg/ml) were added to the medium, respectively. The cultures were maintained for an extra 1–3 h to weaken the cell wall before harvest at 4 °C. Cells were harvested by centrifugation in sealed tubes for 10 min at 6000×*g* and 4 °C. Cells were washed twice with 30 ml ice-cold electroporation buffer and resuspended in a final volume of 1 ml electroporation buffer. Three kinds of electroporation buffers were used, (i) SMP : (0, 270, 500 mM) sucrose, (0, 1) mM MgCl_2_, 5 mM sodium phosphate buffer, pH (6–7.4); (ii) HSM: 5 mM HEPES, 500 mM sucrose, 1 mM MgCl_2_, pH7.4; (iii) SMG: 0.5 mM sorbitol, 0.5 mM mannitol, 10% glycerol. Plasmid DNA (2 μg) was added to pre-chilled electroporation cuvettes (0.2 and 0.4 cm gap, BioRad) followed by 200 μl cell suspension, and the cuvettes were incubated on ice for 10 min.

To optimize electroporation parameters, cells were pulsed under various conditions (6.0–10 kV/cm, 100–400 Ω and 25–50 μF). The optimized parameters were the following: 4–5 ms pulse duration, 7.5 kV/cm (corresponding to 25 μF capacitance and 400 Ω resistance), then electroporated cells were immediately transferred into 5 ml of prewarmed GS medium and incubated at 35 °C for recovery. After overnight culture, all cells were harvested by centrifugation, resuspended in about 100 μl of medium, and plated onto GS agars with 20 μg/ml erythromycin to screen out clones. All manipulations were done in an anaerobic chamber.

### Analysis of promoter activity based on fluorescence intensity

Cell pellets cultivated from 5 ml late-exponential-phase culture in triplicated on each carbon source were washed once with PBS buffer (pH = 7.4, NaCl 137 mmol/L, KCl 2.7 mmol/L, Na_2_HPO_4_ 10 mmol/L, KH_2_PO_4_ 2 mmol/L), then resuspended in 500 μl PBS and disrupted with glass beads (0.1–0.2 mm) using the high-throughput tissue grinder (QIAGEN, Germany). Total protein concentration in the supernatant was estimated using BCA Protein Assay Kit (Sangon, China). Fluorescence intensity was measured by F-4600 fluorescence spectrophotometer (HITACHI, Japan). The excitation and emission wavelengths of FbFP were verified and determined at 452 and 495 nm, respectively, with other parameters set as integration time 5 s, delay time 0.1 s, and PMT Voltage 950 V [14]. This verification was carried out for three times. Finally, fluorescence intensity was normalized by the corresponding total protein concentration for each sample with that of wild-type cells as the baseline.

### Scanning electron microscopy (SEM)

*Clostridium cellulolyticum* and *C. papyrosolvens* were grown anaerobically at 35 °C in 5 ml cellobiose-based GS-2 medium. After 24 h of incubation, the logarithmic phase was reached and the cells were collected (3000–4000 rpm, 5 min). Followed by washing with 1 ml PBS buffer 3 times at the same speed, each sample was incubated with 2.5% glutaraldehyde for 1 h then washed 3 times with PBS buffer for 10 min. Samples are incubated with osmium tetroxide (2%) for 1 h, washed and dehydrated in graded ethyl ethanol (30%–50%–70%–90% each for 5–10 min). Followed by dehydration in anhydrous ethanol (100%) 2 times for 10 min each time, samples were then incubated 10–15 min with a 50:50 [vol/vol] solution of ethanol and tert-butanol and then 100% tert-butanol until complete evaporation and freeze-dried for about 24 h until it is powdered. After gold/palladium alloy coating, samples were observed using a scanning electron microscopy (SEM, S-4800, HITACHI, Japan). The voltage was set at 3.0 kV.

## Additional files

**Additional file 1: Table S1.** RM systems of *C. papyrosolvens*.

**Additional file 2: Figure S1.** Analysis of antibiotic sensitivity of *C. papyrosolvens*. (a) Inhibitory rate of *C. papyrosolvens* was investigated by growing on different types of antibiotics, erythromycin, lincomycin, tetracycline, chloromycetin, apramycin, ampicillin, spectinomycin, hygromycin, and kanamycin. All experiments were performed in triplicate and shown with standard deviations. The curve of inhibitory rate with antibiotic concentration was fitted by the equation of exponential rise to maximum (f = a*(1-b^x)). (b) The 90% inhibitory concentration (IC90) of various antibiotics was determined by the fitted curve and compared.

**Additional file 3: Figure S2.** SEM images of *C. cellulolyticum* (a) and *C. papyrosolvens* (b) cells grown on cellobiose.

**Additional file 4: Table S2.** Primers used in this study.

## Abbreviations

RM: restriction and modification
FbFP: flavin-based fluorescent protein
5′-UTR: 5′ untranslated region
REBASE: Restriction Enzyme Database
MT: methyltransferase
RE: restriction endonuclease
TCS: two-component system
EPS: extracellular polymeric substance
LB: Luria–Bertani medium
IC90: 90% inhibitory concentration
BLAST: basic local alignment search tool

## References

[1] Ren C, Wen Z, Xu Y, Jiang W, Gu Y (2016). *Clostridia*: a flexible microbial platform for the production of alcohols. Curr Opin Chem Biol.

[2] Bayer EA, Belaich JP, Shoham Y, Lamed R (2004). The cellulosomes: multienzyme machines for degradation of plant cell wall polysaccharides. Annu Rev Microbiol.

[3] Bayer EA, Lamed R, White BA, Flint HJ (2008). From cellulosomes to cellulosomics. Chem Rec.

[4] Bayer EA, Shimon LJ, Shoham Y, Lamed R (1998). Cellulosomes—structure and ultrastructure. J Struct Biol.

[5] Maamar H, Abdou L, Boileau C, Valette O, Tardif C (2006). Transcriptional analysis of the *cip*-*cel* gene cluster from *Clostridium cellulolyticum*. J Bacteriol.

[6] Bagnara-Tardif C, Gaudin C, Belaich A, Hoest P, Citard T, Belaich J-P (1992). Sequence analysis of a gene cluster encoding cellulases from *Clostridium cellulolyticum*. Gene.

[7] Celik H, Blouzard JC, Voigt B, Becher D, Trotter V, Fierobe HP, Tardif C, Pages S, de Philip P (2013). A two-component system (XydS/R) controls the expression of genes encoding CBM6-containing proteins in response to straw in *Clostridium cellulolyticum*. PLoS ONE.

[8] Abdou L, Boileau C, de Philip P, Pages S, Fierobe HP, Tardif C (2008). Transcriptional regulation of the *Clostridium cellulolyticum cip-cel* operon: a complex mechanism involving a catabolite-responsive element. J Bacteriol.

[9] Xu C, Huang R, Teng L, Wang D, Hemme CL, Borovok I, He Q, Lamed R, Bayer EA, Zhou J, Xu J (2013). Structure and regulation of the cellulose degradome in *Clostridium cellulolyticum*. Biotechnol Biofuels.

[10] Xu C, Huang R, Teng L, Jing X, Hu J, Cui G, Wang Y, Cui Q, Xu J (2015). Cellulosome stoichiometry in *Clostridium cellulolyticum* is regulated by selective RNA processing and stabilization. Nat Commun.

[11] Blouzard JC, Coutinho PM, Fierobe HP, Henrissat B, Lignon S, Tardif C, Pages S, de Philip P (2010). Modulation of cellulosome composition in *Clostridium cellulolyticum*: adaptation to the polysaccharide environment revealed by proteomic and carbohydrate-active enzyme analyses. Proteomics.

[12] Roberts RJ, Vincze T, Posfai J, Macelis D (2010). REBASE—a database for DNA restriction and modification: enzymes, genes and genomes. Nucleic Acids Res.

[13] Roberts RJ, Vincze T, Posfai J, Macelis D (2015). REBASE—a database for DNA restriction and modification: enzymes, genes and genomes. Nucleic Acids Res.

[14] Teng L, Wang K, Xu J, Xu C (2015). Flavin mononucleotide (FMN)-based fluorescent protein (FbFP) as reporter for promoter screening in *Clostridium cellulolyticum*. J Microbiol Meth.

[15] Ventura JR, Hu H, Jahng D (2013). Enhanced butanol production in *Clostridium acetobutylicum* ATCC 824 by double overexpression of 6-phosphofructokinase and pyruvate kinase genes. Appl Microbiol Biotechnol.

[16] Jennert KCB, Tardif C, Young DI, Young M (2000). Gene transfer to *Clostridium cellulolyticum* ATCC 35319. Microbiology.

[17] Croux C, Nguyen NP, Lee J, Raynaud C, Saint-Prix F, Gonzalez-Pajuelo M, Meynial-Salles I, Soucaille P (2016). Construction of a restriction-less, marker-less mutant useful for functional genomic and metabolic engineering of the biofuel producer *Clostridium acetobutylicum*. Biotechnol Biofuels.

[18] Herman NA, Li J, Bedi R, Turchi B, Liu X, Miller MJ, Zhang W (2017). Development of a high-efficiency transformation method and implementation of rational metabolic engineering for the industrial butanol hyperproducer *Clostridium saccharoperbutylacetonicum* Strain N1-4. Appl Environ Microbiol.

[19] Groom J, Chung D, Olson DG, Lynd LR, Guss AM, Westpheling J (2016). Promiscuous plasmid replication in thermophiles: use of a novel hyperthermophilic replicon for genetic manipulation of *Clostridium thermocellum* at its optimum growth temperature. Metab Eng Commun..

[20] Drepper T, Eggert T, Circolone F, Heck A, Krauß U, Guterl J-K, Wendorff M, Losi A, Gärtner W, Jaeger K-E (2007). Reporter proteins for in vivo fluorescence without oxygen. Nat Biotech..

[21] Mukherjee A, Schroeder CM (2015). Flavin-based fluorescent proteins: emerging paradigms in biological imaging. Curr Opin Biotechnol.

[22] Xu T, Li Y, Shi Z, Hemme CL, Li Y, Zhu Y, Van Nostrand JD, He Z, Zhou J (2015). Efficient genome editing in *Clostridium cellulolyticum* via CRISPR-Cas9 Nickase. Appl Environ Microbiol.

[23] Higashide W, Li Y, Yang Y, Liao JC (2011). Metabolic engineering of *Clostridium cellulolyticum* for production of isobutanol from cellulose. Appl Environ Microbiol.

[24] Nakotte S, Schaffer S, Böhringer M, Dürre P (1998). Electroporation of, plasmid isolation from and plasmid conservation in *Clostridium acetobutylicum* DSM 792. Appl Microbiol Biotechnol.

[25] Lee J, Jang YS, Choi SJ, Im JA, Song H, Cho JH, Seung do Y, Papoutsakis ET, Bennett GN, Lee SY (2012). Metabolic engineering of *Clostridium acetobutylicum* ATCC 824 for isopropanol-butanol-ethanol fermentation. Appl Environ Microbiol..

[26] Tyurin MV, Desai SG, Lynd LR (2004). Electrotransformation of *Clostridium thermocellum*. Appl Environ Microbiol.

[27] Guss AM, Olson DG, Caiazza NC, Lynd LR (2012). Dcm methylation is detrimental to plasmid transformation in *Clostridium thermocellum*. Biotechnol Biofuels.

[28] Cui GZ, Hong W, Zhang J, Li WL, Feng Y, Liu YJ, Cui Q (2012). Targeted gene engineering in *Clostridium cellulolyticum* H10 without methylation. J Microbiol Meth..

[29] Chen L, Lin J, Li B, Lin J, Liu X (2010). Method development for electrotransformation of *Acidithiobacillus caldus*. J Microbiol Biotechnol.

[30] Ferdinand PH, Borne R, Trotter V, Pages S, Tardif C, Fierobe HP, Perret S (2013). Are cellulosome scaffolding protein CipC and CBM3-containing protein HycP, involved in adherence of *Clostridium cellulolyticum* to cellulose?. PLoS ONE.

[31] Gamba P, Jonker MJ, Hamoen LW (2015). A novel feedback loop that controls bimodal expression of genetic competence. PLoS Genet.

[32] Merod RT, Wuertz S (2014). Extracellular polymeric substance architecture influences natural genetic transformation of *Acinetobacter baylyi* in biofilms. Appl Environ Microbiol.

[33] Li YH, Lau PC, Lee JH, Ellen RP, Cvitkovitch DG (2001). Natural genetic transformation of *Streptococcus mutans* growing in biofilms. J Bacteriol.

[34] Rodriguez AM, Callahan JE, Fawcett P, Ge X, Xu P, Kitten T (2011). Physiological and molecular characterization of genetic competence in *Streptococcus sanguinis*. Mol Oral Microbiol..

[35] Desai K, Mashburn-Warren L, Federle MJ, Morrison DA (2012). Development of competence for genetic transformation of *Streptococcus mutans* in a chemically defined medium. J Bacteriol.

[36] Fosses A, Mate M, Franche N, Liu N, Denis Y, Borne R, de Philip P, Fierobe HP, Perret S (2017). A seven-gene cluster in *Ruminiclostridium cellulolyticum* is essential for signalization, uptake and catabolism of the degradation products of cellulose hydrolysis. Biotechnol Biofuels.

[37] Zschiedrich CP, Keidel V, Szurmant H (2016). Molecular mechanisms of two-component signal transduction. J Mol Biol.

[38] Bastet L, Dube A, Masse E, Lafontaine DA (2011). New insights into riboswitch regulation mechanisms. Mol Microbiol.

[39] Cromie MJ, Groisman EA (2010). Promoter and riboswitch control of the Mg^2+^ transporter MgtA from *Salmonella enterica*. J Bacteriol.

[40] Kumar S, Stecher G, Tamura K (2016). MEGA7: molecular evolutionary genetics analysis version 7.0 for bigger datasets. Mol Biol Evol.

[41] Bernhart SH, Hofacker IL, Will S, Gruber AR, Stadler PF (2008). RNAalifold: improved consensus structure prediction for RNA alignments. BMC Bioinformatics.

[42] Lorenz R, Bernhart SH, Honer ZU, Siederdissen C, Tafer H, Flamm C, Stadler PF, Hofacker IL (2011). ViennaRNA Package 2.0. Algorithms Mol Biol..

