# Supplementary material for: Function analysis of 5′-UTR of the cellulosomal xyl-doc cluster in Clostridium papyrosolvens
Source: Biotechnol Biofuels. 2018 Feb 16;11:43. doi: 10.1186/s13068-018-1040-0 (PMC5815224; doi:10.1186/s13068-018-1040-0)
Supplement: Supplementary file 1 — Additional file 1: Table S1. RM systems of C. papyrosolvens. [file 13068_2018_1040_MOESM1_ESM.pdf]

Table S1 RM systems of *C. papyrosolvens*

No.	Name	Coordinates	ORF	Type	Function	Most similar (% identity)
1	M.Cpa2782ORF3030P	51862-53571 c	3030	II	M	M.CceORF2762P (76% identity)
2	M.Cpa2782ORF1545P	88755-91307 c	1545	II	M	M.SscSp3ORF1930002P (65% identity)
3	M.Cpa2782ORF3889P	9878-10795 c	3889	II	M	M.CceORF2787P (89% identity)
4	Cpa2782ORF3872P	311023-314166 c (20254-23397 c)	3872 (2864)	IV	R	Bsp3004ORFKP (57% identity)
5	Cpa2782MrrP	1275-2309 c	2987	IV	R	CpaC7MrrP (94% identity)

M: restriction endonuclease coupled methylation protein; R: restriction endonuclease.
