# Supplementary figures and images for: Function analysis of 5′-UTR of the cellulosomal xyl-doc cluster in Clostridium papyrosolvens
Source: Biotechnol Biofuels. 2018 Feb 16;11:43. doi: 10.1186/s13068-018-1040-0 (PMC5815224; doi:10.1186/s13068-018-1040-0)

a

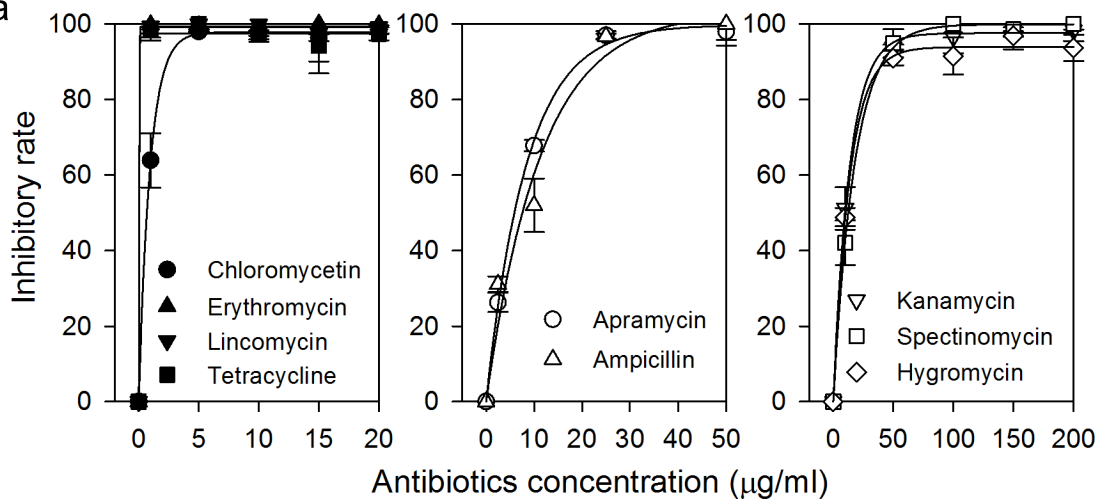

b

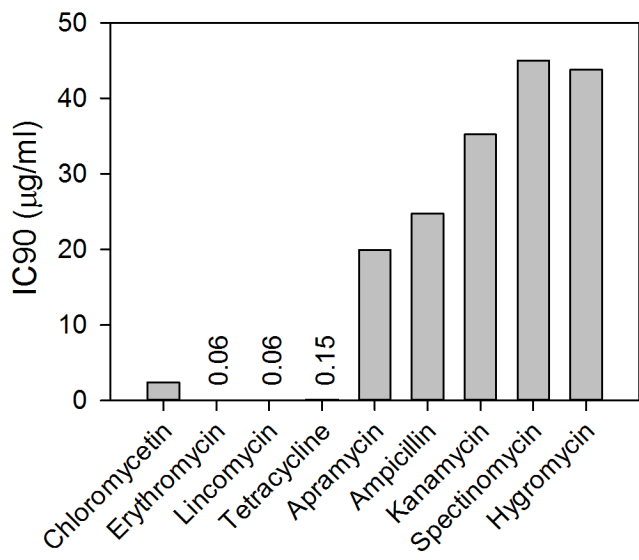

Supplement: Supplementary file 2 — Additional file 2: Figure S1. Analysis of antibiotic sensitivity of C. papyrosolvens. (a) Inhibitory rate of C. papyrosolvens was investigated by growing on different types of antibiotics, erythromycin, lincomycin, tetracycline, chloromycetin, apramycin, ampicillin, spectinomycin, hygromycin, and kanamycin. All experiments were performed in triplicate and shown with standard deviations. The curve of inhibitory rate with antibiotic concentration was fitted by the equation of exponential rise to maximum (f = a*(1-b^x)). (b) The 90% inhibitory concentration (IC90) of various antibiotics was determined by the fitted curve and compared. [file 13068_2018_1040_MOESM2_ESM.pdf]

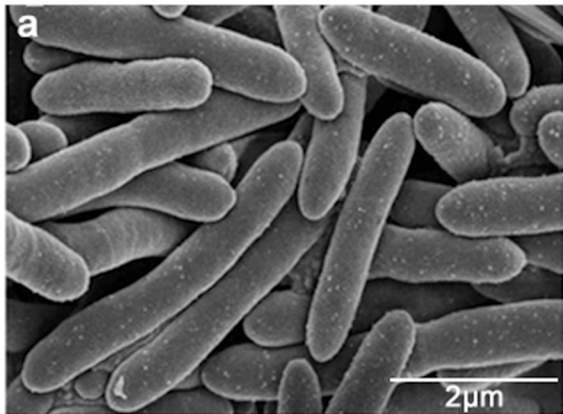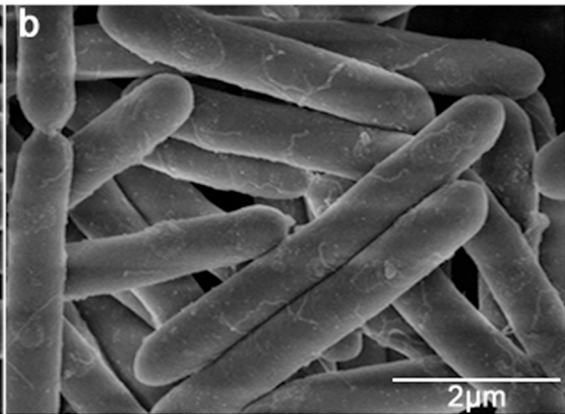

Supplement: Supplementary file 3 — Additional file 3: Figure S2. SEM images of C. cellulolyticum (a) and C. papyrosolvens (b) cells grown on cellobiose. [file 13068_2018_1040_MOESM3_ESM.pdf]
