# Supplementary material for: Function analysis of 5′-UTR of the cellulosomal xyl-doc cluster in Clostridium papyrosolvens
Source: Biotechnol Biofuels. 2018 Feb 16;11:43. doi: 10.1186/s13068-018-1040-0 (PMC5815224; doi:10.1186/s13068-018-1040-0)
Supplement: Supplementary file 4 — Additional file 4: Table S2. Primers used in this study. [file 13068_2018_1040_MOESM4_ESM.pdf]

Table S2. Primers used in this study

Primer name	Primer sequence (5′→3′)	Note
PpFbFPmF	ATGATAAATGCAAAACTTCTTCAGC	To amplify gene *fbfp*
PpFbFPmR	TTAATGTTTTGCCTGACCCTGCTGTC	
P*xyl*_F	TGGCTGCAGCAAAAAAATATATTATAACAAATAG	To generate promoter P*xyl*
P*xyl*_R	CCCTCCGTTTAAATTAATTTTGTAATTATTTTACCATAAG	
UTR$_x$_F	TGGCTGCAGCAAAATATTATACCAATAACAAAAAG	To generate UTR
UTR$_x$_R	TCGACGCGTCATACTTATTTCCCTCCGTTTAAATTAATTT	
P4_F	TGG**C**TGCAGGCTAGCCATAATATATTGACAAATTTATTTTTTAAAG	To generate promoter P4
P4_R	CCCTCCGTTTAAATTAATTTGTAATTATTTTAACTTTAAAAAATAAATTTGTC	
P4UTRx_F	GACAAATTTATTTTTTAAAGTTAAAATAATTACAAAATATTATACC	To generate P4-UTRwith UTRx_R together
